# Unleashing the Power of Synthetic Lethality: Augmenting Treatment Efficacy through Synergistic Integration with Chemotherapy Drugs

**DOI:** 10.3390/pharmaceutics15102433

**Published:** 2023-10-08

**Authors:** Yajing Du, Lulu Luo, Xinru Xu, Xinbing Yang, Xueni Yang, Shizheng Xiong, Jiafeng Yu, Tingming Liang, Li Guo

**Affiliations:** 1 Jiangsu Key Laboratory for Molecular and Medical Biotechnology, School of Life Science, Nanjing Normal University, Nanjing 210023, China; 221202100@njnu.edu.cn (Y.D.); 211202099@njnu.edu.cn (L.L.); 211202068@njnu.edu.cn (X.X.); 221202124@njnu.edu.cn (X.Y.); 2 Department of Bioinformatics, Smart Health Big Data Analysis and Location Services Engineering Lab of Jiangsu Province, School of Geographic and Biologic Information, Nanjing University of Posts and Telecommunications, Nanjing 210023, China; 1022173309@njupt.edu.cn (X.Y.); 1022173308@njupt.edu.cn (S.X.); 3 Shandong Provincial Key Laboratory of Biophysics, Institute of Biophysics, Dezhou University, Dezhou 253023, China; jfyu1979@126.com

**Keywords:** combination therapy, synthetic lethality, PARP inhibitor, ATR inhibitor, WEE1 inhibitor, PRMT5 inhibitor

## Abstract

Cancer is the second leading cause of death in the world, and chemotherapy is one of the main methods of cancer treatment. However, the resistance of cancer cells to chemotherapeutic drugs has always been the main reason affecting the therapeutic effect. Synthetic lethality has emerged as a promising approach to augment the sensitivity of cancer cells to chemotherapy agents. Synthetic lethality (SL) refers to the specific cell death resulting from the simultaneous mutation of two non-lethal genes, which individually allow cell survival. This comprehensive review explores the classification of SL, screening methods, and research advancements in SL inhibitors, including Poly (ADP-ribose) polymerase (PARP) inhibitors, Ataxia telangiectasia and Rad3-related (ATR) inhibitors, WEE1 G2 checkpoint kinase (WEE1) inhibitors, and protein arginine methyltransferase 5 (PRMT5) inhibitors. Emphasizing their combined use with chemotherapy drugs, we aim to unveil more effective treatment strategies for cancer patients.

## 1. Background

With rapid economic development, improvements in quality of life, and changes in risk factors, the epidemiology of cancer in China has undergone significant transformations. Cancer has now become the leading disease in the country. Each year, the National Cancer Center of China approximates the number of newly diagnosed cases to be around 4.064 million. The most prevalent cancer types include lung cancer (20.4%), colorectal cancer (10.0%), stomach cancer (9.8%), liver cancer (9.6%), and breast cancer (7.5%), accounting for a total of 57.3% of all cancer cases [1].

With advances in technology, the five most common cancer treatments to date include physiotherapy–surgical resection, radiotherapy, chemotherapy, targeted therapy, and immunotherapy [2]. Physical therapy, that is, surgical resection, can treat most early benign tumors. However, early tumors are generally not uncomfortable. The majority of cancer cases are diagnosed at intermediate or advanced stages, necessitating the utilization of surgical targeted resection as a crucial modality for cancer treatment. This approach also applies to the management of pancreatic cancer [3,4] and adrenal tumor resection [5]. Radiation therapy is a local treatment that uses radiation to treat tumors, such as lung cancer [6] and breast cancer [7]. However, when killing tumor cells by radiation, normal tissues will also be irradiated with a certain dose, resulting in different degrees of side effects [2]. Chemical drug therapy is chemotherapy, which kills cancer cells by using chemical drugs to achieve the purpose of treatment. It is a systemic treatment and has certain side effects [8]. Targeted therapy can kill systemic cancer cells in a targeted manner [9], and due to its targeting, it has lower side effects than chemotherapy and can be tolerated by most patients, but because of its special mechanism of action, it will cause some cancer cells to develop drug resistance after long-term use [10]. Immunotherapy is a treatment approach that harnesses the body’s immune system to target and eliminate cancer cells and tumor tissues. It works by activating and enhancing the natural immune response against cancer [11].

With the development of genome sequencing, major research investments, and the success of clinical trials, the use of synthetic lethality (SL) strategies to fight cancer has received increasing attention. SL therapy has been called one of the most effective cancer treatments in the past decade [12]. In cancer research, SL is also known as “non-oncogene addiction”, and mutant cancer cells require the activity of SL gene pairs to maintain survival, so targeting the protein products of SL-paired genes would be a good anti-cancer drug target [13]. Compared with normal cells, tumor cells are usually defective in the DNA damage repair pathway, making rapidly proliferating tumor cells more dependent on a specific repair pathway. Therefore, targeting inhibitors of DNA damage repair, such as Poly (ADP-ribose) polymerase (PARP) inhibitors, Ataxia telangiectasia, and Rad3-related (ATR) inhibitors, has become one of the best practices [14]. This study will describe the concept, classification, and screening methods of SL, which can combat most cancer mutations, and combination therapy that can improve the efficacy of chemotherapy drugs [12]. Furthermore, this comprehensive review will explore and find that in cancer treatment, synthetic lethal strategy combined with other drugs can induce the sensitivity of drugs to cancer treatment, so as to achieve better therapeutic effect.

## 2. Synthetic Lethality

### 2.1. The Concept of Synthetic Lethality

**Figure 1:**
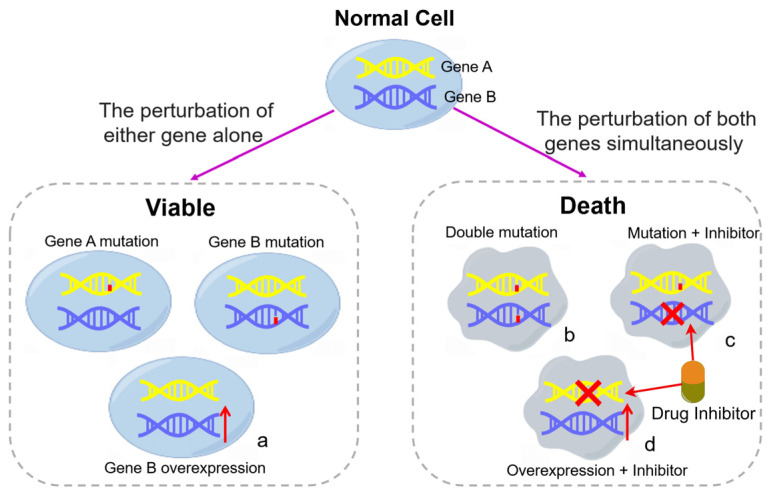
The concept of synthetic lethality (by Figdraw). Cancer cells survive when either gene A or gene B carries a mutation or when gene B is overexpressed (**a**). However, when gene A is mutated, gene B is mutated or inhibited (**b**,**c**), or gene A is inhibited, and gene B is overexpressed (**d**), cells will die due to synthetic lethal interactions. The red part of the DNA double helix structure represents the mutation, while red upward arrows represent gene overexpression. The red fork indicates that the gene is inhibited by drugs.

Synthetic lethality refers to a phenomenon where cells can survive when a single gene is disrupted, but simultaneous disruption of two genes results in cell death. This interaction between the two genes is known as a synthetic lethal interaction [15,16] (Figure 1). This concept was first developed from the genetic study of the model organism *Drosophila melanogaster*, in which it proposed incompatibility between allele pairs and found that when multiple genes are mutated at the same time, cell death can be caused [17,18,19], and the model organism yeast has also contributed to the establishment of the concept of synthetic lethality [20,21].

### 2.2. Synthetic Lethal Classification

Based on the targeting of different gene types, SL can be categorized into synthetic disease lethality and synthetic dose lethality. Additionally, conditional synthetic lethality represents a distinct form of SL [12]. Among these, synthetic disease lethality refers to the scenario where the disruption of a single gene does not impact cell viability, but the simultaneous disruption of two genes results in cell death (Figure 1b). This mechanism can be leveraged to target cancers that are driven by tumor suppressor genes, such as SL of Poly (ADP-ribose) polymerase (PARP) with breast cancer susceptibility gene 1 (BRAC1) [12,22,23]. Synthetic dose lethality is a gene interaction: when one gene is overexpressed, the loss or reduction of the function of the other gene will lead to synthetic lethality. This can be used to target cancers with overexpression of oncogenes, for example, mitotic arrest deficiency 2 (MAD2) and protein-coding phosphatase 2 (PP2A), cyclin-dependent kinase regulatory subunit 1B (CKS1B) and Polo-like kinase 1 (PLK1), KRAS and CDKN1A (p21). MAD2 is a key component of the spindle checkpoint and is overexpressed in many cancer cells. Since the spindle checkpoint pathway is highly conserved between yeast and humans, Yang Bian et al. used yeast screening for synthetic genetic array analysis and found that knocking down the gene PPP2R1A encoding the constant regulatory subunit of PP2A can significantly inhibit the growth of Mad2-overexpressing tumor cells [24]. CKS21B gene is frequently overexpressed in breast cancer, lung cancer, and liver cancer. Reid et al. used high-throughput screening to determine the synthetic dose lethal interaction between CKS1B and PLK1 [25]. KRAS is the main subtype of RAS. KRAS mutations are observed in pancreatic cancer, colorectal cancer, and lung cancer with a particularly high frequency and over-activate downstream pathways. Wang et al. used PLK1 inhibitors and ROCK inhibitors to antagonize cancer cells with KRAS mutations. It was found that this drug combination was mediated by the activation of p21, and it was further found that KRAS and p21 had synthetic dose lethality [26].

### 2.3. Synthetic Lethal Gene Pairs Screening Method

**Figure 2:**
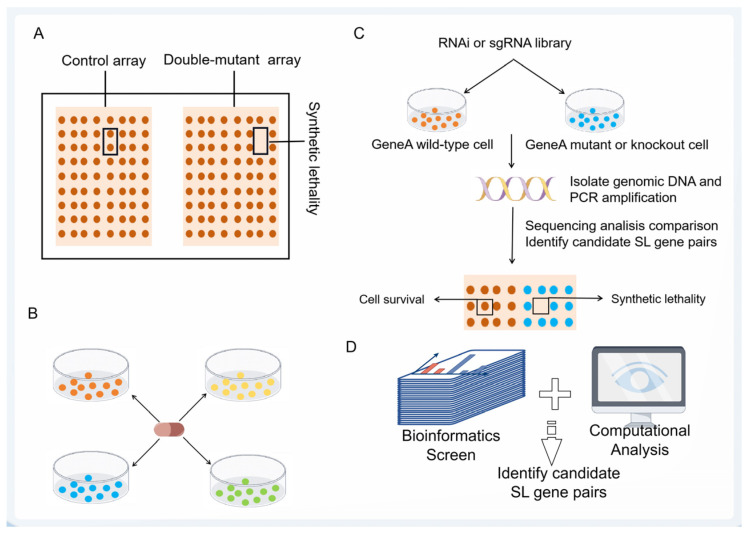
Synthetic lethal screening methods (by Figdraw). (**A**) The synthetic genetic array used a robotic workstation to produce a haploid double mutant yeast by mating and meiotic recombination to identify SL gene pairs. (**B**) Drug screens based on mutation data and high-throughput drug screening data from drug libraries on various cell lines with specific mutations are used to determine the synthetic lethal interaction between drugs and genes. Differently colored circles represent various cell lines with specific mutations. (**C**) RNAi/CRISPR screens were transfected into cells in the form of RNAi or sgRNA library combination for polyclonal screening, then DNA was isolated, PCR amplification was performed, and candidate SL gene pairs were determined by deep sequencing analysis. (**D**) Bioinformatics screens were analyzed at four molecular levels (gene mutation, mRNA expression, methylation, and copy number variation) so as to extract features and use a decision tree model to predict SL gene pairs.

The presence of numerous mutated genes in cancer poses a challenge in identifying thousands of potential SL gene pairs. Several common methods are employed for synthetic lethal screening, including yeast screening, drug screening, RNAi screening, CRISPR screening, and bioinformatics screening (Figure 2) [12].

Yeast screening is the earliest method used to identify SL. It uses yeast for large-scale screening to determine gene pairs with SL [27]. Srivas et al. showcased the effectiveness of yeast screening in narrowing down the pool of potential SL in human cancer cells [28] and that the quantification of genetic interactions and large-scale discovery of double mutants have been greatly facilitated through high-throughput hybridization strategies [29], such as synthetic genetic array analysis (Figure 2A) and microarray synthetic lethal analysis [30]. Although high-throughput yeast mating strategies can improve the efficiency of yeast screening, it remains a challenge to reflect SL gene pairs onto human cancer cells [31]. Drug screening is a method of discovering SL using drug molecules that target specific targets (Figure 2B) [12]. Identification was mainly based on high-throughput drug screening data and cell line mutation data [32]. However, SL screened by this method is highly likely to be less potent and specific than gene knockouts due to insufficient drug dose, side effects, and targeted inhibition [33]. RNAi screening is a technique that utilizes the introduction of exogenous small interfering RNA (siRNA) to specifically knock out target mRNA sequences (Figure 2C). This screening approach can be categorized into two types: short hairpin RNA (shRNA) screening and short interference RNA screening [34,35]. Although the use of RNAi for SL screening has made important contributions to human functional genomics, the utilization of RNAi technology is accompanied by a higher susceptibility to off-target effects, which elevates therapeutic risks and poses limitations on its clinical utilization [36]. CRISPR is a specific, efficient, and scalable genome editing technique that enables complete knockout of target genes for high-throughput screening (Figure 2C) [37]. The advancement of CRISPR technology has enabled the systematic mapping of genetic interactions in human cancer cells. This powerful genetic tool has the potential to greatly accelerate the discovery of SL [38].

Due to the challenges in identifying SL gene pairs and the expensive experimental validation process, computational screening based on biological information has emerged as a crucial method for predicting SL gene pairs (Figure 2D) [39]. Firstly, based on the predicted data of The Cancer Genome Atlas database (TCGA), the four molecular levels of gene mutation, mRNA expression, methylation, and copy number variation can be analyzed so as to extract the features and use the decision tree model to predict the SL gene pairs. For example, the prediction of SL gene pairs for adenomatous polyposis coli (APC) and growth factor Erv1-like gene (GFER) [40].

## 3. Synthetic Lethal Strategy Combined with Other Drugs to Improve Cancer Treatment Effect

Chemotherapy and targeted therapy are important treatments for cancer, but the drugs used in chemotherapy have certain side effects [8], and targeted drugs can develop resistance when taken for a long time [9]. Therapeutic strategies based on SL can be combined with this drug to improve efficacy [12], and several studies have confirmed this conclusion. For example, doxorubicin is the standard of care drug for the early treatment of diffuse large B cell lymphomas (DLBCL) patients. In the study of Salma et al., it was found that Olaparib combined with doxorubicin could inhibit the growth of tumor cells more than doxorubicin alone [41]. Marwan et al. found that the anti-cancer effect of ATR inhibitor AZD6738 combined with chemotherapy on ATM-defective CLL and Mec1 cells was more significant than that of chlorambucil, fludarabine, 4-hydrogen peroxide cyclophosphamide, or Bendamustine monotherapy [42].

### 3.1. The Utilization of PARP Inhibitors in Combination with Other Drugs for Cancer Therapy

The human PARP superfamily comprises 17 known members, which are zinc finger DNA-binding proteins, including PARP1, PARP2, VRARP (PARP4), and end-anchor polymerase 1/2 [43], which can detect and signal DNA single-stranded breaks (SSBs) directly or indirectly produced by genotoxic factors, thereby activating DNA repair [44], of which PARP1 accounts for 90% of cellular DNA repair activity [43]. Upon SSB, the N-terminal zinc finger domain of PARP binds to the damaged DNA, activating the catalytic function of PARP. This leads to the conversion of NAD^+^ to Poly (ADP-ribose) (PAR) and the modification of PARP itself and other proteins. PAR facilitates chromatin remodeling at the site of damage and recruits DNA repair factors such as XRCC1, DNA ligase Ⅲ, DNA polymerase β, and kinases. Through this process, the undamaged DNA strand serves as a template for repair, thereby engaging in the base excision repair (BER) pathway [43,45].

**Figure 3:**
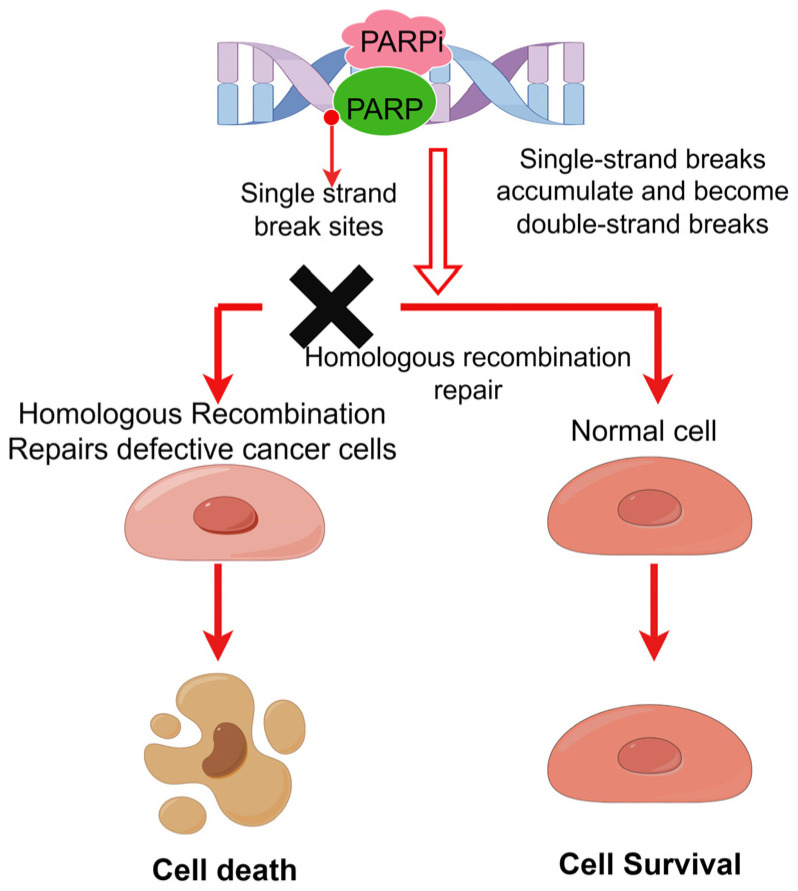
Mechanism of synthesis lethality induced by PARP inhibitors and homologous recombination defects (by Figdraw). PARP inhibitors specifically induce SL in cancer cells with defects in DNA homologous recombination. The black fork indicates that homologous recombination repair cannot be performed.

There are at least five DNA repair mechanisms in cells [46]: homologous recombination (HR), non-homologous end joining (NHEJ), base excision repair (BER), nucleotide excision repair (NER), and mismatch repair (MMR) [47,48]; BER involving the PARP family is the main pathway of the SSB repair system [43]. When the PARP inhibitor is used, PARP enzyme-mediated single-strand break repair pathway is problematic, normal cells can still rely on homologous recombination to repair when the double-strand is broken, while tumor cells are different from normal cells because of their replication and growth characteristics and usually have homologous recombination repair defects (such as BRCA gene mutation [49]); Then, the single-strand break in the tumor cell continues to become a double-strand break, and the double-strand break continues to accumulate, eventually causing cell genome instability and inducing apoptosis. Therefore, PARP inhibitors can selectively kill tumor cells (Figure 3).

PARP inhibitors are cancer therapies that target PARP, and although PARP inhibitors can have a good initial response, prolonged use can make most patients resistant to them, leading to disease recurrence. In recent years, the mechanisms underlying many acquired drug-resistant PARP inhibitors have been described [50,51]. For example, the recovery of BRCA1/2 function leads to HR recovery [52,53], recovery of replication forks [54], mutations of PARP or functionally related proteins [55], and upregulation of drug efflux pumps [56]. Emerging evidence suggests significant promise in combining PARP inhibitors with other targeted drugs, as it creates a synthetic lethal effect by targeting multiple DNA repair pathways simultaneously. For example, some drugs (such as STRIPAK assembly inhibitory peptides 1 and 2, ferroptosis inducer FIN, CSF-1R blocking antibody, and ATR inhibitor) can re-sensitize their cancer to PARP inhibitors, thereby improving the therapeutic efficacy. Striated protein interaction phosphatase and kinase (STRIPAK) complex-mediated inactivation of mammalian STE20-like protein kinases 1 and 2 (MST1/2) increased the DSB repair capacity of cancer cells [57], and the use of STRIPAK assembly to inhibit peptides 1 and 2 (SAIP-1 and SAIP-2) targeting STRIPAK components effectively restored the kinase activity of MST1/2, which was used in combination with PARP inhibitors. Combining PARP inhibitors with other agents can elicit potent synthetic lethality in gastrointestinal tumors. It has been observed that a significant proportion of ovarian cancer patients harbor wild-type BRCA1/2 genes [58], Therefore, PARP inhibitors have no significant clinical benefits, while PARP inhibition or gene deletion can down-regulate the expression of cystine transporter SLC7A11 [59], thereby promoting ferroptosis. Therefore, PARP inhibitors are combined with ferroptosis inducers (FIN) for the treatment of BRCA mature ovarian cancer. Among the extensively studied approaches to target macrophages in anti-cancer therapy, two prominent strategies involve blocking colony-stimulating factor 1 (CSF-1) or its receptor (CSF-1R) to deplete or inhibit tumor-promoting macrophages [60]. Furthermore, combining PARP inhibitors with CSF-1R blocking antibodies has shown considerable potential in augmenting innate and adaptive anti-tumor immune responses [61]. In studies of diffuse large B-cell lymphoma, it was found that the expressed LMO2 protein has functional defects in homologous recombination-mediated DSB repair. LMO2-positive diffuse large B-cell lymphoma is highly sensitive to PARP inhibitors and has a synergistic effect with clinical conventional chemotherapy drugs such as doxorubicin [41]. In prostate cancer, three genes (RNASEH2B, RB1, and BRCA2) are located in close proximity, which are often deleted alone or in combination, resulting in some different clinical outcomes of PARP inhibition [62]. The deletion of RNASEH2B confers sensitivity to PARP inhibition in cancer cells [63,64], when RNASEH2B and RB1 are co-deleted, cells partially lose sensitivity through the expression of BRCA2 induced by E2F1, thereby enhancing homologous recombinant repair ability, ATR inhibition can destroy E2F1-induced BRCA2 expression, so PARP inhibitors combined with ATR inhibitors make it possible to treat tumor patients with RNASEH2B/RB1 co-deletion [62]. Currently, there are several FDA-approved PARP inhibitors, namely Olaparib, Niraparib, Rucaparib, and Talazoparib, that demonstrate significant potential when combined with other drugs in clinical settings [12] (Table 1).

Although several potential mechanisms of resistance to PARP inhibitors have been identified, the majority of clinical data on PARP inhibitors involve their use as second-line therapies. It is possible for multiple tumor subclones in patients to develop resistance to previous treatments, resulting in cross-resistance to PARP inhibitors. This suggests that preclinical research is necessary to investigate resistance mechanisms that differ from those observed in patients. Therefore, gaining a more comprehensive understanding of the role of PARP inhibitors, particularly their involvement in DNA repair, is crucial for advancing our knowledge of PARP inhibitor resistance. This understanding will help maximize the benefits of PARP inhibitor therapy for patients. The combination with targeted drugs may expand the population of patients who benefit, but it is challenging due to the superposition of its toxicity.

### 3.2. Combining ATR Inhibitors with Other Drugs in Cancer Treatment

**Figure 4:**
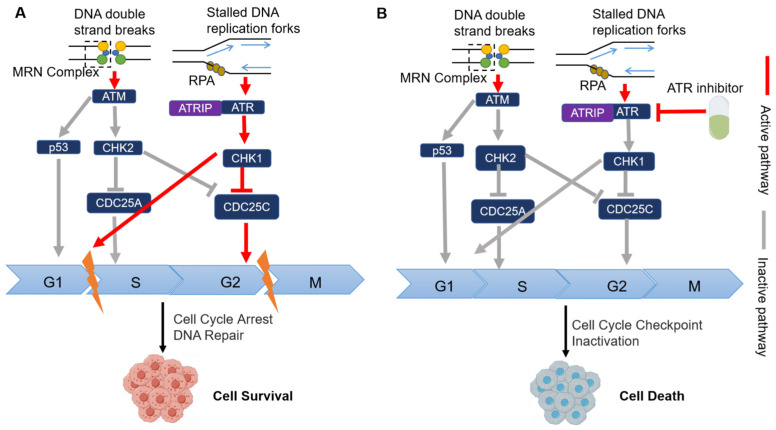
Activation of DNA damage-induced checkpoint signaling pathway in ATM-deficient cells and mechanism of ATR inhibitor and ATM defect-induced synthesis and lethality (by Figdraw). (**A**) DNA double-strand breaks or DNA single-strand breaks activate the ATM/CHK2 and ATR/CHK1 signaling pathways, respectively, and CHK2 and CHK1 phosphorylate CDC25 to eliminate CDK activation, thereby preventing the progression of the cell cycle in the S phase or G2/M phase. (**B**) With ATR inhibitors, ATM-deficient cells die due to cell cycle checkpoint inactivation. Yellow lightning indicates cell cycle arrest. The red arrow indicates that this pathway is activated. The grey arrow indicates that the pathway is inactivated.

When single-stranded DNA (ssDNA) is present due to SSB or replication pressure (RS), replicating protein A (RPA) interacts with the damaged DNA site. Additionally, Ataxia telangiectasia and Rad3-related (ATR) inhibitors bind to ATR interacting protein (ATRIP), and together, they shuttle towards RPA-bound ssDNA, forming the ATR-ATRIP complex [65], and phosphorylation occurs to activate ATR [66,67]. Activated ATR phosphorylates serine/threonine kinase checkpoint kinase 1 (CHK1) [46,68], induced CHK1 phosphorylates cell division cycle 25 (CDC25), and inactivates it [69,70]. Subsequently, cyclin-dependent kinase (CDK) activity is inhibited, leading to the arrest of the S phase and G2/M phase. This cell cycle blockade serves to stabilize replication forks, facilitate DNA repair, and ensure proper DNA maintenance (Figure 4).

Ataxia telangiectasia-mutated gene (ATM) is an SL gene pair with ATR [42]. When a double-strand break (DSB) occurs (Figure 4A), the DNA repair complex MRE11/RAD50/NBS1 (MRN) is recruited to the damaged site, triggering the autophosphorylation and activation of ATM [71,72]. Activated ATM then phosphorylates CHK2, leading to the inactivation of CDC25 phosphatase. As a result, CDK activity is inhibited, causing cell cycle arrest [73,74]. In cancer, the loss of G1 checkpoint control is commonly observed, making cancer cells heavily reliant on the activation of S and G2/M phase checkpoints mediated by ATR/CHK1 signaling [69]. ATM-deficient tumor cells rely more on the ATR/CHK1 signaling pathway than normal cells, so when ATR inhibitors are used, they will cause SL to tumor cells (Figure 4B).

In preclinical studies, the combination of ATR inhibitors with platinum (Pt) drugs has enhanced the therapeutic effect of Pt-based chemotherapy in cancer, such as VX-970 combination with cisplatin, which demonstrates complete inhibition of tumor growth in three models that are insensitive to cisplatin, as well as long-lasting tumor regression in cisplatin-sensitive models [75]. In esophageal squamous cell carcinoma, VX-970 makes cancer cells significantly sensitive to cisplatin, especially ATM-deficient cells [76]. AZD6738 can be used in combination with cisplatin in squamous cell carcinoma of the head and neck, improving sensitivity to cisplatin [77]. ATR inhibitors, in combination with PARP inhibitors, can be used as a means of overcoming resistance to PARP inhibitors. NU6027 sensitizes BRCA wild-type MCF7 breast cancer cells to PARP inhibitors [78]. The combination of ATR inhibitors and PARP inhibitors exhibited superior effectiveness compared to PARP inhibitors alone in reducing tumor burden in recurrent BRCA mutation models [79]. In a BRCA1-mutant ovarian cancer cell model resistant to acquired PARP inhibitors, Burgess et al. found that the ATR inhibitor VE-821 in combination with Olaparib worked better than Olaparib alone [80]. Most ATR inhibitors take a combination strategy in clinical studies, consistent with a large number of preclinical studies supporting ATR inhibitors as sensitizers for DNA damage chemotherapy and PARP inhibitors (Table 2). ATR inhibitors currently entering the clinical stage include AZD6738, BAY1895344, VX-970, VX-803, M1774, ATRN-119, and RP-3500. The structural formula of the first four inhibitors have been disclosed; the last four structural formulas have not been disclosed [81]. The combination of VX-970 and topotecan in the treatment of platinum-refractory small-cell carcinoma was first reported [82]. In advanced solid tumors, the combination of VX-970 with cisplatin or gemcitabine demonstrated favorable tolerability and showed promising preliminary clinical activity, providing compelling evidence for progressing into phase II trials [83,84]. In 2021, Kim et al. reported that AZD6738, in combination with paclitaxel, exhibited good tolerability in the treatment of patients with advanced malignant tumors [85]. Furthermore, Jo et al. proposed that VX-803 exhibited a highly synergistic effect with various clinically relevant replication stress inducers, such as topotecan and irinotecan, in the treatment of patient-derived tumor organoids [86].

Despite the demonstrated synergistic anti-cancer effects of combining ATR inhibitors with chemotherapy and targeted drugs in various types of cancer, both in preclinical and clinical studies, the development of ATR inhibitors with therapeutic efficacy continues to pose challenges. Combination therapy has garnered significant attention in the pharmaceutical industry as a potential strategy, yet the development of effective ATR inhibitors remains a complex task. ATR kinases are structurally very similar to other PIKK family members, which makes the selectivity of ATR inhibitors a challenge. In a phase II trial conducted in patients with metastatic urothelial carcinoma, the addition of VX-970 to cisplatin in combination with gemcitabine did not result in survival prolongation. Additionally, a trend of reduced survival was observed [87].

### 3.3. Combining Drugs with WEE1 Inhibitors for Cancer Treatment

**Figure 5:**
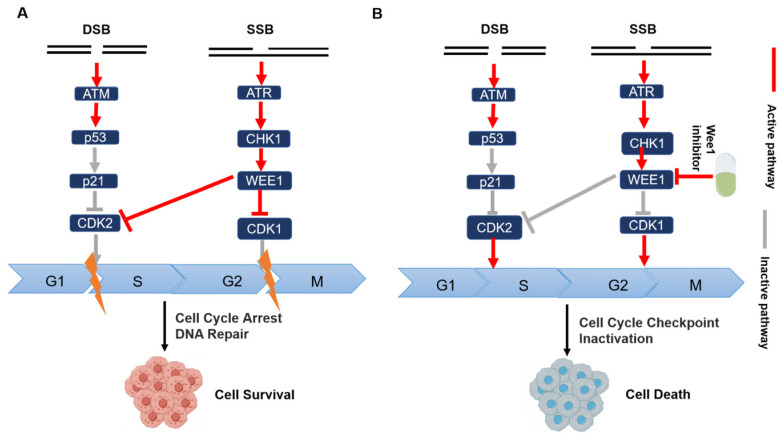
WEE1 and TP53 synthetic lethal interaction mechanism (by Figdraw). (**A**) In cancer cells with TP53 mutation, when SSB occurs, ATR can activate CHK1, and the activated CHK1 can activate WEE1, thereby inhibiting the activity of CDK1 and CDK2, arresting the cell cycle in G1/S and G2/M phases, and obtaining more time for cells to repair DNA damage. (**B**) After using WEE1 inhibitors in cancer cells with TP53 mutations, the blocking effect disappears, and the cells will die due to the mitotic catastrophe. Yellow lightning indicates cell cycle arrest. The red arrow indicates that this pathway is activated. The grey arrow indicates that the pathway is inactivated.

WEE1 kinase, a crucial member of the serine/threonine protein kinase family, is categorized as a cell cycle regulatory protein [88]. When a single-strand break occurs in DNA (Figure 5), the ATR signaling pathway becomes activated, leading to the simultaneous phosphorylation of CDC25C and WEE1 by activated CHK1. WEE1 kinase can block the process of cellular mitosis through two pathways, namely S-phase and G2/M phase arrest, to gain time for repairing DNA damage [89]. The p53 protein plays a critical role in G1 checkpoints, and mutations in the *TP53* gene result in impaired G1 checkpoint mechanisms in numerous cancer cells. Consequently, these cells become more reliant on DNA repair processes regulated by G2/M checkpoints [90]. The use of WEE1 inhibitors can abolish the G2/M checkpoint, and its application to TP53 mutant tumors will cause SL. Liang et al. reported that WEE1 inhibitors can also selectively inhibit stem cell carcinomas and gliomas with ATRX mutations [91]. Lewis et al. found that in cervical cancer and breast cancer cell lines, AZD1775, in combination with paclitaxel, reduced cell survival [92]. WEE1 inhibitors sensitize triple-negative breast cancer and cisplatin-resistant cancer cells to cisplatin because inhibition of WEE1 can more profoundly damage DNA replication checkpoints and the loss of G2/M checkpoints [93]. Hirai et al. reported that AZD1775 enhanced the cytotoxicity of doxorubicin, 5-fluorouracil, Camptothecin, and other DNA damage agents in vitro [94]. Preclinical trials support the combination of AZD1775 with ATR inhibitors or PARP inhibitors to produce anti-tumor effects [95,96]. These preclinical studies support the use of WEE1 inhibitors in combination with other drugs as a combination strategy against tumors. In clinical trials (NCT00648648), AZD1775 was combined with gemcitabine, cisplatin, and carboplatin for the treatment of advanced solid tumors [97], and paclitaxel in combination with carboplatin for ovarian cancer, fallopian tubes, and primary peritoneal tumors with P53 mutations (NCT01357161). The safety and tolerability of ZN-c3 in combination with carboplatin, macrogol liposomal doxorubicin, paclitaxel, and gemcitabine for the treatment of platinum-containing chemotherapy-resistant or refractory ovarian cancer is currently being evaluated in a phase I clinical trial (NCT04516447). Additionally, the use of Debio0123 in combination with carboplatin for the treatment of recurrent or refractory locally advanced or metastatic solid tumors is currently in stage I of development (NCT03968653).

Small molecule inhibitors, including AZD1775, ZN-c3, and Debio0123, are progressing into clinical trials. Combination therapy holds more promise than monotherapy, as it allows for lower doses of WEE1 inhibitors and enables treatment optimization through intermittent administration, thereby improving drug tolerance. However, the clinical toxicity and side effects associated with these inhibitors remain a significant concern. For instance, the combined use of AZD1775 with chemotherapy has demonstrated toxicity that limits its clinical development, and a reliable biomarker for this combination therapy is currently lacking. Ongoing research explores the combination of AZD1775 with targeted therapy and immunochemotherapy. Although the combination strategy involving AZD1775 has not yet been thoroughly investigated, there are still numerous possibilities for combining WEE1 inhibitors with other targeted drugs.

### 3.4. Combining Drugs with PRMT5 Inhibitors for Cancer Treatment

Protein arginine methyltransferase 5 (PRMT5) belongs to the family of protein arginine methyltransferases (PRMTs). As an epigenetic enzyme, PRMT5 plays a crucial role in modifying various substrates, including both histone and non-histone proteins. One of the main substrates for PRMT5 methylation is p53, which is able to regulate cell cycle arrest as well as apoptosis [98]. In addition to regulating the cell cycle, PRMT5 is involved in transcriptional regulation, RNA metabolism, and ribosome biosynthesis [99]. Upregulation of PRMT5 expression has been observed in various malignancies, including lung cancer, ovarian cancer, colorectal cancer, breast cancer, and melanoma. This upregulation has been associated with a poor prognosis for patients with these cancer types [100].

**Figure 6:**
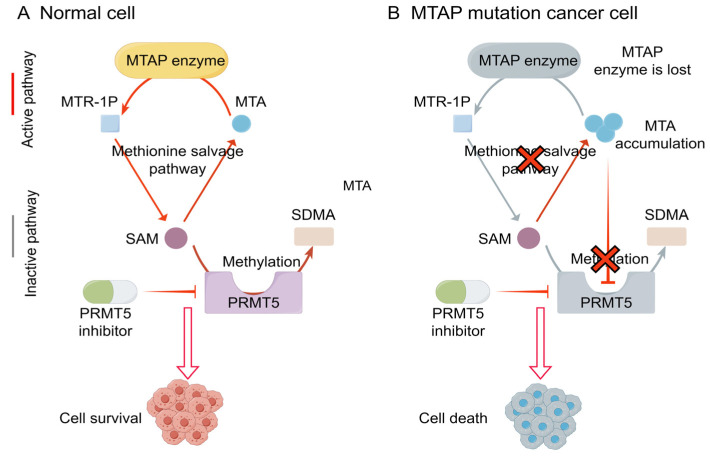
The mechanism map of the synthetic lethality interaction between PRMT5 and MTAP (by Figdraw). (**A**) In normal cells, MTAP is active and able to convert MTA to SAM by methionine salvage pathway. SAM acts as a substrate for PRMT5 to form SDMA through methylation, and PRMT5 inhibitors can relatively reduce methylation. (**B**) In MTAP-mutant cancer cells, MTAP is inactivated and fails to catalyze MTA, causing excessive accumulation of MTA in the cells. At this time, MTA competes with the substrate SAM of PRMT5, inhibits the activity of PRMT5, and leads to increased sensitivity to PRMT5 depletion. If PRMT5 inhibitors are used, cell death will occur. The red arrow indicates that the reaction is activated. The grey arrow indicates that the reaction is inactivated.

The SL gene chaperone of PRMT5 is methyl methylthionine phosphorylase (MTAP) (Figure 6) [101,102]. MTAP is a tumor suppressor gene that is a key enzyme in the methionine and purine synthesis rescue pathway, catalyzing the production of methionine by adenosine methylthionine (MTA), which is essential for maintaining normal cell function. However, it is often co-deleted with CDKN2A and the proportion of this phenomenon in tumors can reach 9–15% [103]. When the *MTAP* gene is lost, its substrate MTA accumulates, and the accumulation of MTA competes with the substrate of PRMT5, S-adenosyl-L-methionine (SAM), which reduces PRMT5 activity, and the loss of MTAP and the accumulation of MTA lead to sensitivity to PRMT5 inhibition [101].

Gemcitabine is a first-line chemotherapy agent for the treatment of pancreatic ductal adenocarcinoma (PDAC) and is the backbone of several drug combinations for PDAC patients. CRISPR screening was used to determine that inhibition of PRMT5 in an orthotopic patient-derived xenograft model (PDX) can make PDAC cells develop a synergistic vulnerability to gemcitabine, thereby improving the therapeutic efficacy of existing chemotherapy for PDAC [104]. Tadalafil, as an inhibitor of PRMT5, increases breast cancer susceptibility to doxorubicin by reducing RNA m6A methylation [105]. In their study, Khuloud et al. demonstrated that the combination of arginine methyltransferase inhibitor 1 (AMI-1) with cisplatin resulted in a significant reduction in viability and induction of apoptosis in lung adenocarcinoma cells. Importantly, the use of AMI-1 did not affect normal bronchial epithelial cells [106]. T-1-44, as a PRMT5 inhibitor in combination with TGF-β1 signaling inhibitor Vactosertib, significantly reduces pancreatic cancer tumor size and prolongs survival [107]. In mouse melanoma models, GSK3326595, in combination with PD-1 inhibitors, significantly reduced tumor size and significantly increased survival compared with PRMT5 inhibitors or PD-1 inhibitors alone [108].

In clinical trials, the combination of AMG193 and docetaxel in the treatment of advanced MTAP-null solid tumors entered stage II (NCT05094336). Another trial (NCT03614728) is evaluating the safety and efficacy of GSK3326595 in combination with 5-azacitidine for recurrent and refractory myelodysplastic syndrome (MDS) and chronic myelomonocytic leukemia. PRMT5 inhibitors, as an emerging class of anti-tumor drugs, are currently in the early stages of clinical development. They have demonstrated broad therapeutic potential in clinical trials as monotherapies for the treatment of solid tumors and hematologic malignancies, as seen in trials such as NCT04089449 and NCT03886831. Furthermore, several studies have indicated that combination therapy involving PRMT5 inhibitors can significantly enhance the anti-tumor efficacy and overcome drug resistance caused by chemotherapy drugs. Ongoing trials aim to further investigate the safety and reliability of such combination therapies.

### 3.5. Combining Drugs with DNA-PK Inhibitors for Cancer Treatment

DNA-dependent protein kinase (DNA-PK), a serine/threonine protein kinase complex, is a holoenzyme complex composed of DNA-binding catalytic subunits (DNA-PKcs) and KU70/80 heterodimers. It is the largest protein kinase in the phosphatidylinositol 3-kinase-related kinase (PIKK) family [14]. The main function of DNA-PK is to repair DSB, which plays a key role in non-homologous end joining (NHEJ) [109,110]. Cells have evolved two different DSB repair pathways, including homologous recombination (HR) and NHEJ. HR is a relatively slow but error-free pathway that relies on homologous sequences present in the genome as templates to replace damaged DNA fragments [111]. Unlike HR, NHEJ is a relatively fast but inherently error-prone process. Since NHEJ does not require DNA template for repair, different nucleases and ligases are used to directly connect its ends, making it error-prone [112,113].

**Figure 7:**
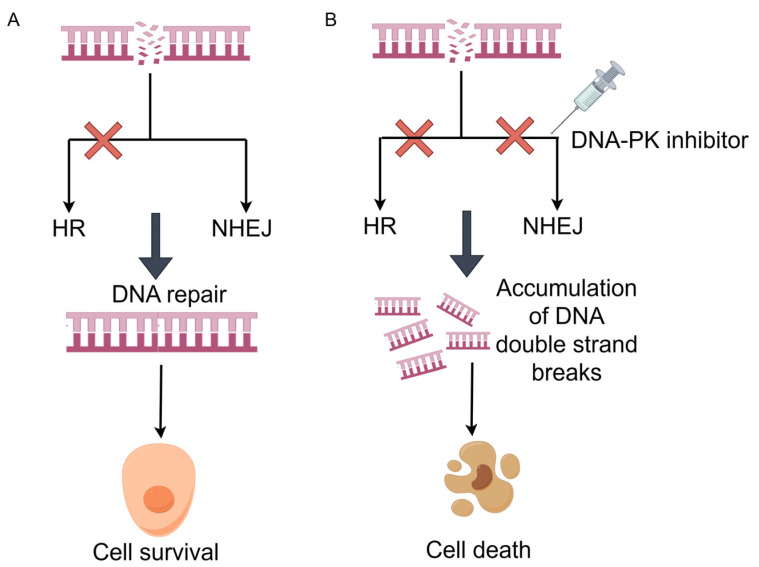
DNA-PK synthesis lethal mechanism (by Figdraw). (**A**) In cancer cells with HR defects, DSB can be repaired through the NHEJ pathway. Finally, the cells can survive. (**B**) When DNA-PK inhibitors are used in cancer cells with HR defects, the NHEJ pathway is destroyed, and the DSB in the cells is continuously accumulated, eventually leading to cell death. The red fork indicates that the corresponding DNA damage repair pathway is destroyed.

DNA-PK, BRCA1 [114], ATM [115], and other genes that can participate in the HR pathway constitute the SL gene pair (Figure 7). The main mechanism is that the HR and NHEJ pathways are damaged, and DSB continues to accumulate, eventually leading to cell death. For peposertib, as an inhibitor of DNA-PK, which has drug resistance in TP53 mutant tumor cells, Jeffrey et al. found that POLQ (polymerase θ, POL θ) and DNA-PK constitute synthetic lethal interaction in the microhomology-mediated end-joining pathway, so combining POL θ inhibitor with DNA-PK inhibitor could significantly improve the efficacy of TP53 mutant solid tumors [116]. The DNA-PK inhibitor AZD7648 is an effective sensitizer of doxorubicin and, in ATM-deficient cells, in combination with Olaparib, enhanced the efficacy of Olaparib, enabling sustained tumor regression [117]. NU7441, a DNA-PK inhibitor, was able to enhance the sensitivity of NSCLC to topoisomerase inhibitors by blocking DNA damage repair [118]. In clinical studies, AZD7648 combined with cytotoxic drugs/novel anti-cancer drugs have entered phase I/a (NCT03907969). CC-115 has conducted phase I clinical trials for glioblastoma to evaluate the efficacy (NCT02977780) in combination with Temozolomide, Neratinib, and QBS10072S. The combination of M3814 and Avelumab in solid tumors has entered stage I (NCT03724890). Clinical trials of other DNA-PK inhibitors such as BR101801, NU7026, and other inhibitors in combination with other drugs need further investigation.

## 4. Conclusions and Prospect

Tumor cells are characterized by their high mutation rates and genomic instability, often accompanied by impaired DNA stability and repair mechanisms. Traditional cancer treatments mainly rely on inducing DNA damage through cytotoxic agents such as alkylating agents, topoisomerase inhibitors, and mitotic spindle inhibitors [119]. However, tumor cells can develop resistance to chemotherapy drugs through genetic mutations, such as *TP53* gene mutations, enabling them to continue replicating despite DNA damage [119]. The concept of SL has emerged as a promising strategy in targeted DNA damage repair. It involves the inhibition of specific genes to disrupt the DNA repair function of cancer cells, ultimately triggering apoptosis. Combining SL strategies with chemotherapy drugs can further enhance DNA damage, thereby increasing cancer cells’ sensitivity to chemotherapy drugs and overcoming drug resistance.

Although the combination of SL and chemotherapy has shown promise in preclinical and clinical studies, several challenges remain in cancer treatment. Accurately identifying and validating potential synthetic lethal interactions in different genetic contexts is a significant challenge. Furthermore, the development and optimization of DNA damage repair inhibitors and the management of cross-talk and negative feedback in DNA damage pathways pose additional complexities. The combination of SL strategies with other chemotherapy drugs may introduce unknown therapeutic effects or toxic side effects, necessitating thorough clinical research to determine optimal treatment sequences and dosages. In conclusion, combination therapy holds great potential as an effective treatment modality, expanding the scope of benefits and providing more treatment options for patients.

## Figures and Tables

**Table 1:** Clinical trials of combination therapy with PARP inhibitors.

PARP Inhibitor	Combined Drugs	Cancer Type	Clinical Stage	ClinicalTrials.gov Identifier	First Posted Date	Status
Olaparib (Patent number: US-201802 15741-A1)	Abiraterone	Prostate cancer	Ⅲ	NCT03732820	November 2018	Active
	Pembrolizumab	Cervical cancer Metastatic melanoma Metastatic colorectal cancer	Ⅱ Ⅱ Ⅱ	NCT04641728 NCT04633902 NCT05201612	November 2020 November 2020 January 2022	Active Recruiting Recruiting
	Temozolomide	Triple-negative breast cancer Colorectal cancer	Ⅱ	NCT04166435	November 2019	Completed
	Durvalumab	Metastatic pancreatic cancer Epithelial ovarian cancer	Ⅱ Ⅱ	NCT05659914 NCT04644289	December 2022 November 2020	Recruiting Recruiting
	Bevacizumab	Recurrent small-cell lung cancer	Ⅱ	NCT04939662	June 2021	Recruiting
	Selumetinib	Advanced or recurrent solid tumors	Ⅰ	NCT03162627	May 2017	Active
Niraparib (Patent number: US-1045 7680-B2)	Dostarlimab	Ovarian cancer Head and neck cancer	Ⅲ Ⅱ	NCT03602859 NCT04068753	July 2018 August 2019	Active Recruiting
	Bevacizumab	Recurrent endometrial or ovarian cancer	Ⅱ	NCT05523440	Augyst 2022	Recruiting
	Trastuzumab	Metastatic HER2-positive breast cancer	Ⅰ/Ⅱ	NCT03368729	December 2017	Recruiting
Rucaparib (Patent number: US-1120 2782-B2)	Nivolumab	Biliary tract cancer High-grade serous or endometrial ovarian cancer	Ⅱ	NCT03639935 NCT03824704	August 2018 January 2019	Active Terminated
	Copanlisib	Prostate cancer	Ⅰ	NCT04253262	February 2020	Active
Talazoparib (Patent number: US-113115 37-B2)	Avelumab	Metastatic renal cell carcinoma Breast cancer	Ⅱ Ⅰ/Ⅱ	NCT04068831 NCT03964532	August 2019 May 2019	Active Active
	Nivolumab	Metastatic or unresectable melanoma	Ⅱ	NCT04187833	December 2019	Active
	Sacituzumab Govitecan	Breast cancer	Ⅰ/Ⅱ	NCT04039230	July 2019	Recruiting
	Pinaru	Metastatic castration-resistant prostate cancer	Ⅰ	NCT05425862	June 2022	Recruiting

**Table 2:** Clinical trials of ATR inhibitor combination therapy.

ATR Inhibitor	Registered Patent	Combined Drugs	Cancer Type	Clinical Stage	ClinicalTrials.gov Identifier	First Posted Date	Status
VX-970	US-20150 359797-A1)	Gemcitabine	Ovarian primary peritoneal or fallopian tube cancer	Ⅱ	NCT02595892	August 2016	Active
		Irinotecan	Gastric cancer, gastroesophageal junction cancer	Ⅱ	NCT03641313	November 2020	Active
		Topotecan	Small-cell lung carcinoma	Ⅰ/Ⅱ	NCT02487095	July 2015	Active
		Cisplatin	Squamous cell carcinoma of the head and neck	Ⅰ	NCT02567422	September 2016	Active
AZD6738	US-2020 0147077-A1	Olaparib	Platinum-refractory extensive small-cell lung cancer	Ⅱ	NCT02937818	November 2016	Active
		Durvalumab	Non-small-cell lung cancer	Ⅱ	NCT03334617	December 2017	Active
		Paclitaxel	Advanced solid tumors	Ⅰ	NCT02630199	December 2015	Completed
BAY1895344	US-20190 022176-A1	Niraparib	Advanced solid tumor, ovarian cancer	Ⅰ	NCT04267939	February 2020	Recruiting

## Data Availability

Not applicable.

## Abbreviations

APC	Adenomatous polyposis coli
ATM	Ataxia telangiectasia mutant gene
ATRIP	ATR-interacting protein
BER	Base excision repair
BRAC1	Breast cancer susceptibility gene 1
CDC25	Cell division cycle 25
CHK1	Checkpoint kinase 1
CHK2	Checkpoint kinase 2
DSB	Double-strand break
DLBCL	Diffuse large B cell lymphomas
GFER	Growth factor ERv1-like genes
HR	Homologous recombination
MAD2	Mitotic arrest deficient protein 2
MMR	Mismatch repair
MTA	Methylthionine
MTAP	Methylthionine phosphorylase
NER	Nucleotide excision repair
NHEJ	Non-homologous end joining
PAR	Poly (ADP-ribose)
PARP	Poly ADP-ribose polymerase
PDAC	Pancreatic ductal adenocarcinoma
PDX	Patient-derived xenograft model
PP2A	Protein phosphatase 2A
PRMT5	Protein arginine methyltransferase 5
Pt	Platinum
RS	Replication pressure
SL	Synthetic lethality
SSB	Single-stranded breaks
